# Automatic classification and segmentation of single-molecule fluorescence time traces with deep learning

**DOI:** 10.1038/s41467-020-19673-1

**Published:** 2020-11-17

**Authors:** Jieming Li, Leyou Zhang, Alexander Johnson-Buck, Nils G. Walter

**Affiliations:** 1 Single Molecule Analysis Group, Department of Chemistry and Center for RNA Biomedicine, The University of Michigan, Ann Arbor, MI USA; 2 Department of Physics, The University of Michigan, Ann Arbor, MI USA; 3 Department of Internal Medicine, Division of Hematology/Oncology, University of Michigan, Ann Arbor, MI USA; 4 Present Address: Bristol-Myers Squibb Company, New Brunswick, NJ USA; 5 Present Address: Google, Pittsburgh, PA USA

**Keywords:** Total internal reflection microscopy, Software, Single-molecule biophysics, Machine learning

## Abstract

Traces from single-molecule fluorescence microscopy (SMFM) experiments exhibit photophysical artifacts that typically necessitate human expert screening, which is time-consuming and introduces potential for user-dependent expectation bias. Here, we use deep learning to develop a rapid, automatic SMFM trace selector, termed AutoSiM, that improves the sensitivity and specificity of an assay for a DNA point mutation based on single-molecule recognition through equilibrium Poisson sampling (SiMREPS). The improved performance of AutoSiM is based on accepting both more true positives and fewer false positives than the conventional approach of hidden Markov modeling (HMM) followed by hard thresholding. As a second application, the selector is used for automated screening of single-molecule Förster resonance energy transfer (smFRET) data to identify high-quality traces for further analysis, and achieves ~90% concordance with manual selection while requiring less processing time. Finally, we show that AutoSiM can be adapted readily to novel datasets, requiring only modest Transfer Learning.

## Abstract (web summary)

Traces from single-molecule fluorescence microscopy (SMFM) experiments exhibit photophysical artifacts that typically make analysis time-consuming. Here, the authors have developed an easily accessible software, AutoSiM, for two distinct applications of deep learning to the efficient processing of SMFM time traces.

## Introduction

Single-molecule fluorescence microscopy (SMFM) is a powerful family of approaches with applications in biophysics, analytical chemistry, and super-resolution microscopy^1–4^. For instance, smFRET is a widely used technique to measure small-scale distance changes (typically in the range of ~2 to 8 nm) by detecting changes in the efficiency of FRET over time in each molecule or complex. This approach permits the observation of equilibrium biomolecular dynamics that would be inaccessible to ensemble techniques^5^. In addition, kinetic fingerprinting approaches such as single-molecule recognition through equilibrium Poisson sampling (SiMREPS)^4,6^ employ kinetic probing to achieve highly specific detection of single unlabeled nucleic acids. SiMREPS in particular has been shown to distinguish between a wild-type DNA sequence and C-to-T point mutation^7^ with an apparent discrimination factor several orders of magnitude greater than the theoretical maximum for methods relying on thermodynamic discrimination^8^.

**Fig. 1:**
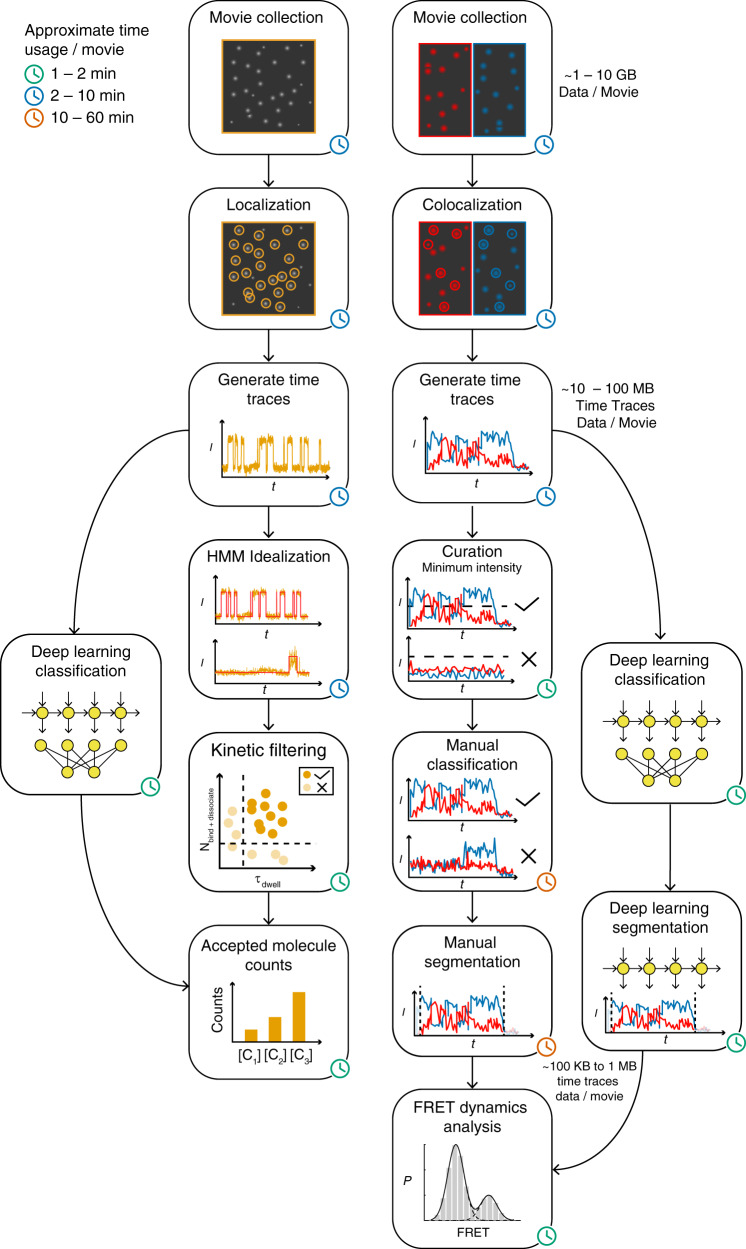
Data analysis pipelines for single-channel SiMREPS (left) and two-channel smFRET (right) SMFM time traces as implemented in AutoSiM. The steps in each pipeline that are bypassed by the deep learning methods in this paper are shown. The approximate time required per movie for each step of analysis is indicated by a green, blue, or orange clock icon. In the chart, *I* = luorescence intensity, *t* = time, *P* = probability, *N*_bind+dissociate_ = the number of observed binding and dissociation events per trace, and *τ*_dwell_ = dwell time.

Single-molecule fluorescence microscopy data are usually acquired using a wide-field total internal reflection fluorescence (TIRF) microscope with sensitive EMCCD or sCMOS camera(s) (Fig. 1). The raw movie data are saved and stored in an uncompressed format that takes ~1–10 GB per movie, or perhaps 10–100 GB per experiment. Emission profiles from single fluorophores are localized within the field of view by various spot-finding methods to identify candidate molecules for analysis^9–11^. In the case of two-channel measurements such as most smFRET experiments^9^, the signals from the two fluorophores are spectrally separated using a dichroic mirror and projected onto different zones of the camera’s sensor, or onto different cameras, and fluorescent signals originating from the same molecule must be paired up (or colocalized) using one of several image registration methods^12–14^. Next, an intensity-versus-time trace is generated for each candidate molecule, reducing the size of a typical dataset to ~10–100 MB/movie.

Analysis of intensity-*versus*-time traces varies depending on the nature and goal of the experiment (Fig. 1). For single-channel SiMREPS experiments, HMM^15^ is frequently used to fit an idealized (noiseless) time trace to the noisy raw data in order to extract kinetic information from each time trace; this information is then subjected to a thresholding procedure to identify which candidate traces exhibit kinetic behavior (i.e., frequency and lifetimes of probe binding events) within the range expected for the analyte of interest. The traces passing the kinetic thresholding step are classified as Accepted, and the number of accepted traces is taken as the number of analyte molecules observed in the field of view. By contrast, for two-channel smFRET data (Fig. 1) first a curation step is used to screen out traces with low fluorescence intensity and/or signal-to-noise ratio to avoid the calculation of inaccurate FRET values; this can be performed in automated or semi-automated fashion by simple thresholding. After curation, researchers typically must manually select time traces of sufficiently high quality to accurately reflect the FRET dynamics of the experimental system. Following this manual classification step, only the segment(s) of each trace in which both fluorophores are capable of continuous excitation and emission (i.e., are not blinking or irreversibly photobleached) must be selected for analysis in order to avoid the calculation of spurious FRET values. This segmentation is often performed manually as well, although automated segmentation algorithms are available^16^. The resulting dataset of segmented, high-quality smFRET traces has usually dwindled to between ~100 KB and 1 MB per movie, and is then subjected to further analyses (e.g., HMM and/or construction of FRET histograms) that depend on the specific questions under investigation.

The power of many SMFM methods thus comes at the price of a need for extensive data curation and analysis. In some cases, such as super-resolution microscopy, data analysis has been automated with satisfactory results^17–20^. However, in other cases, such as smFRET and SiMREPS, existing algorithms for single-molecule trace selection usually result in trade-offs between false positives and false negatives depending on the values of various arbitrary thresholds set by the user. This limitation is due to the sheer diversity of potential artifacts, making it difficult to design simple criteria that effectively remove all artifactual or defective time traces while retaining all or most of the relevant data for further analysis. To mitigate this tradeoff, most laboratories manually screen hundreds to thousands of single-molecule traces prior to analysis (Fig. 1), but this will require lengthy training to become an expert, introduces potential user-dependent error that will likely evolve with training, and may still consume several person-hours of effort per experiment (Supplementary Fig. 1). There is thus a great need for faster and more accurate automated analytic pipelines for SMFM time traces that can manage the complexities of single-molecule behavior.

Several groups have developed software tools that automate some aspects specifically of smFRET analysis^16,21–23^. One of the most advanced and full-featured of these programs is SPARTAN^16^, which automates the curation and segmentation steps of analysis. However, thresholding parameters for curation are set by hand in a trial-and-error process, and the user must still manually select high-quality traces from the curated set, which can be time-consuming for datasets containing thousands of traces. In addition, conventional algorithms for characterizing the kinetic behavior of traces introduce significant errors and biases. For instance, a regularly used method for kinetic analysis in SMFM—HMM^15^—will occasionally mistake the tail of a noisy intensity or FRET signal as an additional state, resulting in spurious kinetic transitions. While methods such as hierarchical clustering of HMM-fitted smFRET trajectories permit automated classification of even complex single-molecule behaviors^24^, these methods are only as accurate as the original fitting method. For an analytical technique such as SiMREPS, even an occasional error can have a strong effect on specificity and limit of detection^25^. Finally, it is often the case that only a particular time segment of each single-molecule trace (e.g., prior to photobleaching of the first fluorophore) is useful; these regions of interest (ROIs) are often selected by hand, slowing analysis considerably.

In recent years, deep learning has provided innovative solutions for single-molecule analysis, with applications ranging from the acceleration of super-resolution microscopy^19,26^ to identification of single molecules in noisy fluorescence microscopy images^27,28^, improvement of the accuracy of nanopore-based DNA sequencing^29^ and classification of kinetic single-molecule barcodes for multiplexed microscopy^30^. Here, we adapt a recurrent neural network (RNN) known as Long Short-Term Memory (LSTM)^31^ to the analysis of experimental SiMREPS datasets where the experimental ground truth (presence or absence of a mutant DNA sequence spiked into the matrix) is known, and show that the LSTM algorithm yields consistently higher sensitivity and specificity than conventional HMM followed by application of signal-to-noise ratio and kinetic thresholds. Furthermore, we compare LSTM with a convolutional neural network (CNN)^32^ for the classification and segmentation analysis of single-molecule FRET traces, and show that the concordance between the classifications made by users and the algorithms remains high (~90%). Finally, to facilitate the application of our deep learning networks to new SMFM systems we implement Transfer Learning, achieving high concordance (~90%) for a distinct smFRET-monitored biomolecular system without the need for a large training dataset. Our results suggest that deep learning approaches provide a valuable means of both accelerating and improving the classification and parsing of single-molecule time trajectories for biophysics and analytical chemistry. We make our software, named AutoSiM, available upon request as a MATLAB executable, allowing end users with no machine learning background to easily apply it to their own data.

## Results

### An LSTM classifier improves analysis of SiMREPS time traces

**Fig. 2:**
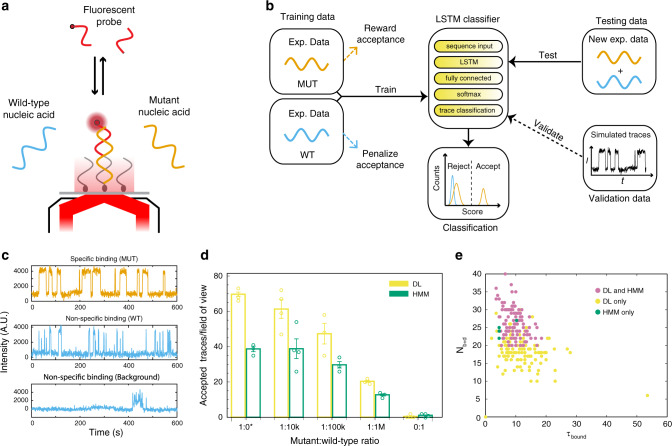
Training and classification using the LSTM deep learning algorithm of AutoSiM for the analysis of one-color single-molecule traces with known ground truth. **a** Schematic of experimental system for detection of a mutant DNA sequence by SiMREPS. **b** Schematic of LSTM data processing. In the training phase, since the presence of even a few false positives (MUT detection events) in the WT dataset has a strong impact on specificity, the training loss function strongly penalizes the classification of traces from the WT dataset as MUT traces. In the validation phase, we used simulated traces to understand the network’s decision. Validation is optional for routine use. In the testing phase, we examined the accuracy of classification by the trained LSTM network on an independent dataset comprising mixtures of MUT and WT sequences at varying ratios. **c** Representative experimental SMFM traces showing repeated binding to mutant molecule, as well as a typical trace showing non-repetitive nonspecific binding (including wild-type-like and background-like). **d** Comparison of the number of SMFM traces accepted as evidence of mutant DNA sequences by LSTM deep learning (DL) and HMM + kinetic thresholding (HMM).* 1:0 condition is from training data and other four conditions are from testing data. Open circles represent individual experimental replicates, bars represent the mean of all replicates for a given condition, and error bars represent 1 S.E.M. with *N* = 4 for 1:10k; *N* = 3 for 1:0 (MUT only), 1:100k, and 1:1 M; and *N* = 8 for 0:1 (WT only). **e** Comparison of apparent kinetic parameters *N*_b+d_ and *τ*_bound_ for traces accepted by DL and HMM methods. The DL method accepted all traces that were accepted by HMM + kinetic thresholding except for a small minority (shown in green).

As a challenging data analysis case that provides a useful experimental ground truth against which to judge the performance of the algorithm, we tested the potential of deep learning to improve the analysis of SiMREPS data for detection of the EGFR mutation T790M^18^ (Fig. 2). The SiMREPS assay distinguishes among surface-immobilized mutant and wild-type DNA sequences on the basis of different kinetics of probe interaction with the mutant sequence, wild-type sequence, and the surface itself. Distinguishing the T790M mutation from wild-type EGFR sequence at low mutant allele fractions is especially challenging because it is a C → T transition that is susceptible to interference from spontaneous deamination of C to yield U^33^. The time traces of mutant and wild-type detection events are potentially very similar and therefore, due to the stochasticity inherent in single-molecule kinetics, challenging to distinguish perfectly using a simple thresholding algorithm. We assigned two labels to training datasets according to experimental conditions: Mutant for experiments in which the T790M sequence is spiked into the matrix, and Wild-Type for experiments in which only wild-type EGFR sequence is present. Mutant datasets yield many SiMREPS traces with repeated transitions between high- and low-fluorescence states, indicating the presence of repeated specific binding of a fluorescent probe to the mutant sequence (Fig. 2a, c). By contrast, wild-type datasets comprise time traces exhibiting a range of different behaviors that reflect partially mismatched binding of probes to wild-type DNA sequence (Fig. 2a, c). Both mutant and wild-type datasets contain additional time traces reflective of nonspecific probe binding to other molecular species or to the imaging surface.

We trained the LSTM classifier of AutoSiM entailing 5 neural network layers (Fig. 2b) on a training dataset combining 3 mutant and 4 wild-type experiments, with 2184 time traces in total. The objective of training was to (1) abstract time-dependent signal features that are highly represented in the mutant dataset, while poorly represented in the wild-type dataset; and (2) use these features to assign a score to individual traces that classifies each trace as a likely mutant or wild-type/non-specific-binding detection event. During training, we used a weighted cross-entropy loss function that weakly rewards the acceptance of traces from the mutant training datasets while strongly penalizing the acceptance of traces from the wild-type training datasets (Fig. 2b). The output of the LSTM network is a probability score ranging from 0 (wild-type-like or background-like) to 1 (mutant-like), representing the likelihood that each trace represents probe binding to a mutant molecule. Based on the assigned score, the LSTM network classifies each trace as accepted (mutant-like) or rejected (wild-type-like or background-like) (Fig. 2 and Supplementary Fig. 2). An ensemble histogram of scores assigned to traces in the training datasets (Supplementary Fig. 3a) shows that the wild-type dataset exhibits scores clustered near 0, while the mutant dataset contains two well-separated populations with high and low scores, showing high specificity and certainty, with very few boundary cases to complicate the classification task. By default, any trace with a score >0.5 is classified as a positive mutant detection event, and any trace with a score <0.5 is classified as likely wild-type or background signal. Inspection of scores assigned to specific traces in the training mutant dataset (Supplementary Fig. 2) finds that high-scoring traces tend to show clear repetitive transitions between two states and good signal-to-noise ratio, reflecting the distinct time-dependent signal features present only in mutant detection events. In contrast, low-scoring traces have disqualifying features such as more than two intensity states, few transitions between high- and low-intensity states, or binding events that occur with kinetics that are very different than those observed for the mutant DNA sequence. Notably, no traces in the wild-type dataset have scores above 0.5. While training requires 30–60 min on a typical laptop computer, this is hands-off time, in contrast to the manual adjustment of HMM filtering parameters that may take a similar amount of time but is a hands-on process strongly influenced by the skill of the experimenter. Application of the trained model to any future dataset (comprising, in our test below, the classification of 5457 single-molecule traces) takes ~2 min on a laptop computer.

To further understand the basis of the score assignment, we applied the LSTM classifier to simulated data with randomized intensity and kinetic parameters, and found that both signal-to-noise ratio and kinetics strongly factor into the classification of a trajectory, consistent with the most critical differences between true positive mutant traces and background traces (Supplementary Fig. 3b–d). Notably, unlike the conventional kinetic analysis based on HMM, the LSTM classifier does not apply hard thresholds to individual kinetic parameters, but makes classifications based on the simultaneous consideration of multiple coupled parameters. These results illustrate the capability of AutoSiM to learn the underlying time-dependent features of SiMREPS time traces without any a priori assumptions about binding kinetics.

Important to the robust application of any machine learning method is the ability to maintain high performance on new, independent datasets. After training, we tested the trained LSTM classifier on independent experimental data from detection of the T790M mutation in the presence of a varying excess of wild-type sequence^25^ (Fig. 2c). SiMREPS experiments conducted with four different ratios of mutant sequence to wild-type sequence (1:10k, 1:100k, 1:1 M and 0:1) were included, comprising 14 experiments and 5,457 traces in total. Compared to classification based on HMM fitting followed by kinetic thresholding, the LSTM algorithm consistently accepts approximately 1.7-fold as many traces in the presence of mutant sequence (1:10k, 1:100k and 1:1 M), yielding a ~70% increase in sensitivity, while accepting 2.5-fold fewer traces (2 accepted in LSTM classifier vs. 5 accepted in the HMM fitting followed by kinetic thresholding) in the absence of mutant sequence (0:1), suggesting a ~4.25-fold increase in specificity (Fig. 2d). The LSTM classifier also increased the sensitivity of a standard curve for the T790M sequence in the absence of wild-type sequence (Supplementary Fig. 4). We note that in the training set with only mutant sequence (1:0) the LSTM approach also accepted more traces than the HMM approach, while in the training set with only wild-type sequence (0:1) the LSTM network accepted fewer traces than the HMM approach. Taken together, these results suggest that AutoSiM can robustly abstract the underlying time-dependent features with high certainty, and can improve the effective limit of detection while also increasing analytical specificity from the same underlying raw data.

### Deep learning accelerates analysis of smFRET time traces

**Fig. 3:**
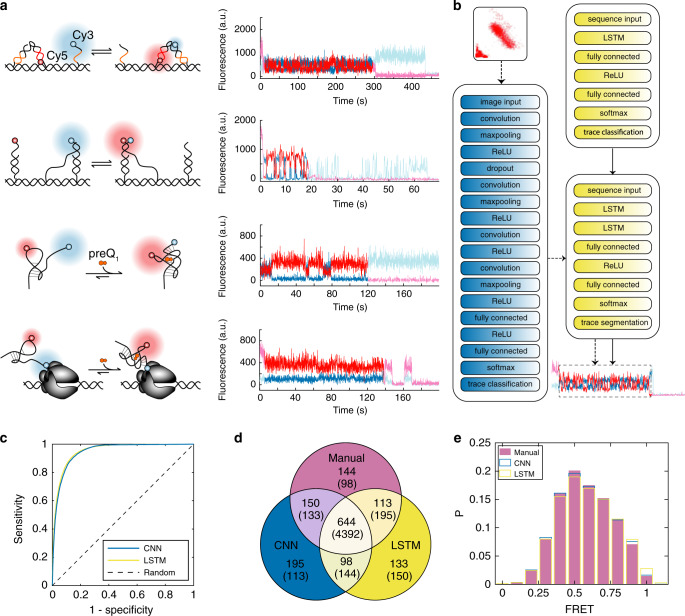
Classification and segmentation of two-color single-molecule FRET traces with the LSTM and CNN deep learning algorithms of AutoSiM. **a** Schematics and typical smFRET traces from the four experimental systems used in training and testing, from top to bottom in the panel: a toehold-exchange-based DNA walker^37^; **a** DNA swinging arm^36^; a preQ_1_ riboswitch^39^; and a paused transcriptional elongation complex^38^. Traces show variable numbers of anticorrelated changes in donor (blue) and acceptor (red) fluorescence intensity typical of smFRET data. The elevated acceptor fluorescence from 0 to 5 s in some of the traces is due to direct excitation of the acceptor to confirm its presence prior to the FRET measurement. **b** LSTM network structures for classification and segmentation (blue) and CNN network structure (yellow). The LSTM network accepts raw SMFM traces for training and testing, while the CNN network requires that each trace be converted into a 2-D scatterplot of donor and acceptor intensity, then into a 32-by-32-pixel image for processing by the CNN. **c** ROC curves for classification by the CNN and LSTM networks as compared to classifications by human experimenters. Here, specificity and sensitivity are calculated in relation to manual selection outcomes rather than a known ground truth. AUC > 0.95 for both networks, indicating strong agreement with manual classification. **d** Venn diagram showing the concordance among manual selection, LSTM and CNN for a representative test set of single-molecule trace data. Numbers in the Venn diagram represent the number of traces accepted (rejected) by each method or combination of methods. **e** FRET histograms generated by CNN-classified, LSTM-classified, and manually selected traces from the same two original datasets. Data used is from a paused transcriptional elongation complex experimental system.

Since its development over two decades ago^1^, smFRET has been used to study the dynamics of many biomolecular systems at the nanometer scale, especially conformational changes involving proteins^34,35^ and/or nucleic acids^36–39^ (Fig. 3a). In these studies, the need to simultaneously consider the behavior of two fluorescence channels (i.e., donor and acceptor fluorophores) and the lack of an objective experimental ground truth (e.g., presence or absence of probe binding to a mutant sequence) by which to judge the output of analysis makes this a more complex task for an automated classifier than SiMREPS. Since the most time-consuming tasks in smFRET for most laboratories are the curation, classification, and segmentation of smFRET time traces (Fig. 1), we developed a deep learning-based component of AutoSiM that automates these steps for two-channel smFRET traces. Since the LSTM neural network is sensitive to time-dependent signal changes, it can be trained to perform not only binary curation and classification of traces (Accept or Reject) but also segmentation to exclude portions of traces exhibiting photobleaching or photoblinking^40^ or other sporadic events that interfere with FRET analysis (Supplementary Fig. 5). Both classification and segmentation can therefore use the same LSTM network design with user-defined network structure parameters such as the preferred network depth that best reflects the given data complexity and desired prediction granularity. For this application of AutoSiM (Fig. 3b), we used a 7-layer LSTM network (Supplementary Table 11) for classification based on user-assigned labels (Accept or Reject, one label for each trace), and an 8-layer LSTM network (Supplementary Table 12) for segmentation based on the segment(s) of each trace that were selected or rejected for analysis by the user (i.e., one label for each frame in the time trace).

To train these networks with a broad range of FRET dynamics, we compiled a smFRET dataset composed of 122 raw movies from four different users and four distinct molecular systems^36–39^ (Fig. 3a), selected 80% of the traces at random for training, and reserved the remaining 20% of traces for evaluation. The combined dataset consists of 5110 distinct traces that were accepted by the users and 24,285 traces that were rejected, representing ~140 person-hours of manual classification and segmentation. To further expand the range of possible FRET dynamics beyond those observed in this limited number of molecular systems, the training dataset was supplemented with 5000 simulated Accepted traces with randomized FRET states and interconversion kinetics. Since there are no objective expectations for the output of analysis based on an experimental ground truth as with SiMREPS data, we use the concordance—calculated as the ratio of the number of traces agreed upon by both human and the classifier algorithm divided by the total number of input traces—as the performance metric for these smFRET data. For the classification LSTM network, an average concordance of 90.2 ± 0.9% was achieved for 10 training and test sets sampled at random from the same experimental dataset comprising 122 raw movies (Supplementary Tables 1 and 2). For the segmentation LSTM network, an average frame-to-frame concordance of ~95% was achieved. In contrast to the pace of 1–5 traces processed per minute by human users (Supplementary Fig. 1), the trained LSTM networks classify ~1000 traces per minute and perform segmentation of approximately 1,000 traces per minute on a desktop with Intel i5 3.4 GHz CPU, accelerating analysis 100- to 500-fold while maintaining high concordance with human experimenters.

A hallmark of FRET measurements is to evaluate the degree of anticorrelation between donor and acceptor fluorophore emission intensity within each single-molecule trace. To directly consider this feature, we converted the two-channel time series data from each trace into a two-dimensional scatter plot of donor and acceptor intensity values, which was then formatted as an image. These images, whose features reflect not only any correlation between donor and acceptor intensity within each trace but also the number of FRET states, signal-to-noise ratio, photobleaching etc., were used for training and evaluation of a CNN implemented in AutoSiM (Supplementary Table 13), thus converting the classification task into one of image recognition (Fig. 3b). Using the same sampling scheme for training (80% of data) and evaluation (20% of data) as was used for the LSTM classifier, an average concordance of 90.7 ± 0.4% with human experimenters was achieved across 10 trials with different randomly sampled training and evaluation sets (Supplementary Tables 3 and 4). Although the CNN yields slightly higher concordance than the LSTM classifier, it requires more computing power than that of a typical laptop computer (all the CNN training in this work was performed using GPUs from Amazon Cloud). To avoid the necessity of cloud computing, our software allows users to employ our pre-trained CNN for classification purposes without the need to comprehensively train a new network. Like the LSTM network, the trained CNN performs classification much more rapidly than human users, processing ~8000 traces per minute on a desktop with Intel i5 3.4 GHz CPU.

To more comprehensively evaluate the concordance of these two classification networks with human judgments as a function of score threshold, we constructed receiver-operator characteristic (ROC) curves (Fig. 3c). The area under the curve (AUC) for both the LSTM and CNN curves is ~0.95, suggesting high proficiency at predicting the classification choices of a human experimenter. The concordance between LSTM, CNN, and manual trace selection for a representative test set of data is shown as a Venn diagram in Fig. 3d. The overall specificity of the LSTM (94.6 ± 1.0%) and CNN approaches (94.5 ± 0.6%) is comparable, and can be increased further (97.9 ± 0.3 %) by accepting only those traces that are accepted by both methods (Supplementary Tables 1–6), albeit at the expense of lower sensitivity (57.4 ± 4.5%) relative to manual selection. In addition, FRET histograms generated by one human user and by the automatic trace selectors (CNN+LSTM) are shown in Fig. 3e, and suggest reasonable agreement between the output of the deep learning classifiers and human experimenters. FRET histograms for the other three users and systems show reasonable agreement as well (Supplementary Fig. 6). Detailed analysis of traces accepted by the LSTM algorithm but not manual selection (Supplementary Fig. 7) or vice-versa (Supplementary Fig. 8) suggests that both approaches are fairly conservative, rejecting traces that may in fact yield useful FRET data but contain small imperfections such as low signal-to-noise ratio or intensity changes in one channel without a corresponding anticorrelated change in the other channel.

Taken together, we so far have demonstrated that deep learning-based algorithms can automate and bypass the most time-intensive steps of smFRET data analysis—manual classification and segmentation—while exhibiting high concordance with human researchers for a broad range of molecular systems. These approaches not only significantly accelerate data analysis but, perhaps more importantly, also are expected to provide more consistent results than human judgment because the underlying algorithms are deterministic. A potential shortcoming is that, as with other deep learning approaches, ours requires a large training set: to train the networks on as broad a range of FRET dynamics and data artifacts as possible, we included 29,395 manually analyzed time traces for training and evaluation. Requiring such a large training set for analysis of new smFRET systems would render the approach impractical. However, as we show in the next section, most of the network layers trained with this broad dataset can be reused in the analysis of new molecular systems, which significantly reduces the amount of training data required when generalizing the method to new datasets.

### Adaptation to new smFRET datasets with minimal training data

**Fig. 4:**
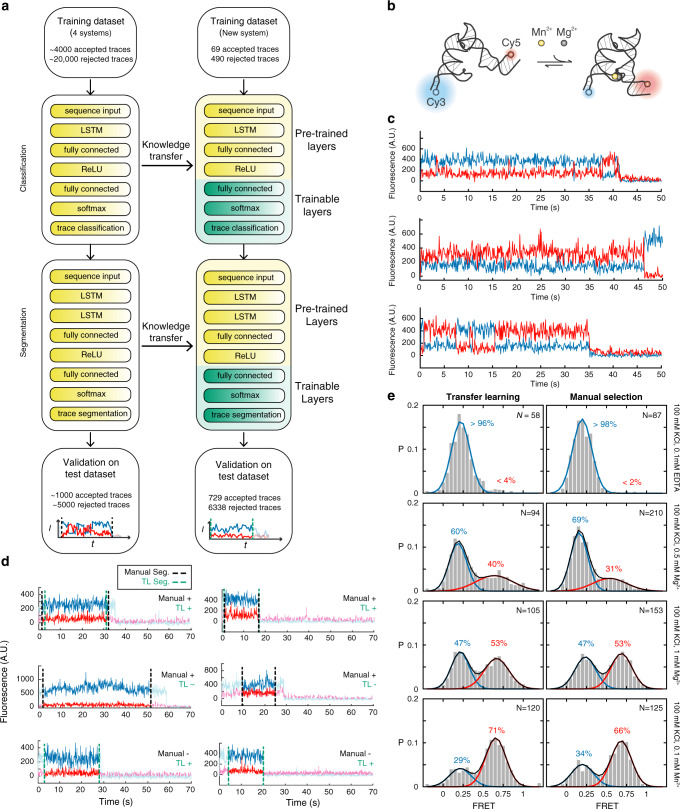
Adaptation of previously trained networks to a novel smFRET system with Transfer Learning by AutoSiM. **a** Schematic depiction of TL. Networks previously trained on a large database of smFRET traces are adapted to a new system by retraining only the final layers of each network using a small training set from the new experimental system. **b** Schematic of a previously characterized Mn^2+^ riboswitch system^42^ that is used here as a test case for TL, since data from this system were not present in the original training set for the LSTM networks. **c** Three representative time traces from the Mn^2+^ riboswitch system illustrating its diverse molecular behaviors: static low-FRET (top), static high-FRET (middle), and dynamic (bottom). **d** Representative traces illustrating True Positive, False Negative, and False Positive classification and segmentation results from TL. **e** FRET histograms for the Mn^2+^ riboswitch following classification and segmentation by TL or manual selection, with two-peak Gaussian fits and corresponding population estimates for the low- and high-FRET states. The number of traces (N) included is shown in the upper right corner of each histogram.

To facilitate the application of our deep learning approach to novel smFRET systems without requiring large training datasets, we exploit Transfer Learning (TL). TL is a training strategy used in deep learning that transfers knowledge learned from one problem (dataset) to a different but related problem (dataset) by reusing most of the trained network layers^41^. In our AutoSiM software, we transferred the first 4 layers of the 7-layer classification LSTM network (149,800 parameters, representing 99.97% of the total parameters) and first 5 layers of the 8-layer segmentation LSTM network (342,625 parameters, representing 99.98% of the total parameters) to analyze data from a molecular system not included in the original training set, a Mn^2+^-sensing riboswitch^42^ (Fig. 4b). During training, we fixed the parameters in the transferred layers while only training the last 3 layers of the networks (52 parameters, representing 0.02% and 0.03% of the total parameters in the classification and segmentation networks, respectively). With the benefit of transferred layers being trained with broad FRET dynamics, the training was accomplished within 15 min using only 559 manually analyzed time traces (equivalent to the number of time traces generated from 1 raw movie, comprising 69 manually accepted and 490 manually rejected traces) from the new dataset, only 2.3% of the size of the original smFRET training set comprising ~24,000 traces. Notably, we used data only from a single experimental condition (100 mM KCl, 0.1 mM Mn^2+^) that exhibited a broad range of possible molecular trace behaviors (Fig. 4c).

After training, the networks were applied to test data comprising 15 experimental movies and 7146 traces from the same system under five different buffer conditions resulting in different FRET dynamics. On average, the networks achieved 91% concordance with manual classification (Supplementary Table 7), and FRET histograms constructed for each buffer condition excluding the training condition were compared to corresponding histograms from manual selection (Fig. 4e). Two-peak Gaussian fitting revealed similar estimates of the population of the high- and low-FRET (docked and undocked, respectively) states across buffer conditions, with a discrepancy between fitted FRET population ranging from <1% to 9%. Differences of this magnitude routinely arise when comparing replicates of the same experiment collected and analyzed on different days by the same user, and do not affect the overall conclusion^42^ that the docked state of this riboswitch is promoted by higher concentrations of Mg^2+^ and especially Mn^2+^. Closer inspection of the output reveals that, for all four conditions tested, the TL-trained network accepted fewer time traces than manual selection. This is expected because the network is optimized to yield high concordance with the training data (Supplementary Table 7); since the majority of traces are rejected in the training set, high concordance generally requires the network to be conservative in its selections. Inspection of the individual traces classified differently by the TL-trained network than by manual selection reveals that these are often borderline cases in which the signal-to-noise ratio or stability of the fluorescent signal intensity are poor but perhaps still high enough for an accurate FRET measurement, and which are often judged inconsistently even by the same user (Fig. 4d). In contrast, as a deterministic classifier, the LSTM network makes more consistent judgements over time and between experiments than an individual human user, increasing the consistency between experiments compared to manual classification.

To compare our software with existing state-of-art software, we tested the same Mn^2+^-sensing riboswitch dataset with SPARTAN^16^, which can automate trace segmentation but not classification with user-defined threshold values for a list of software-calculated features (Supplementary Table 10). To provide a direct comparison with our approach, we used the same data (559 traces, 69 manually accepted and 490 manually rejected) from the same buffer condition (100 mM KCl, 0.1 mM Mn^2+^) as in the training of LSTM network to manually search for a set of threshold values that classifies the traces in high concordance with manual selection. We then applied the threshold values to the same data used in testing the TL-trained networks (comprising 7146 traces), which yielded a curated set of traces exhibiting on average 86% concordance with manual selection (Supplementary Table 8), somewhat lower than that observed for the TL-trained network. Note that SPARTAN provides a functionality for users to manually refine classification results and manually segment traces before subsequent analysis of FRET dynamics by inspecting the curated traces individually. Assuming the user can perform the analysis at 5 traces per min, the highest continuous pace among the four users we surveyed (Supplementary Fig. 1), the total user time to generate the necessary output for analysis of FRET dynamics for all the testing data is ~4 h with SPARTAN (Supplementary Table 9), only ~16% of the time required for manual analysis. By comparison, the LSTM network of AutoSiM both classified and segmented the test data at a rate of 1,000 traces per min, leading to a total analysis time of ~15 min (~1 min per movie), only ~1% of the time required for manual analysis without any fatigue; that is, ~16 times faster than SPARTAN.

In summary, we have demonstrated that deep learning algorithms can be robustly adapted to the analysis of new SMFM data with TL. We trained the LSTM networks of AutoSiM on training data equivalent to those from only one raw movie from one experimental buffer condition from a new biomolecular system with diverse molecular trace behaviors, and applied the trained network to test data comprising 15 experimental movies from the same system under 5 experimental buffer conditions. High concordance (91%) with manual selection was achieved and the same overall experimental conclusions could be drawn as from previous manual analysis^42^. AutoSiM not only greatly accelerates smFRET data analysis and provides higher concordance with manual analysis than current state-of-the-art software, but is also expected to provide more consistent output than manual analysis.

## Discussion

We here have developed two applications of deep learning to the processing of SMFM time traces. We show that our software AutoSiM can (1) reduce the time required for analysis; (2) improve analytical performance in the case of SiMREPS data; and (3) achieve high concordance with human selection in the case of smFRET. Our results demonstrate the generalizability and transferability of trained deep learning networks to new data through (1) the application of a single-channel LSTM classifier to a SiMREPS dataset collected in the presence of varying ratios of T790M mutant-to-wild-type DNA sequence, as well as a T790M standard curve; and (2) the application of two-channel LSTM classification and segmentation networks to an smFRET dataset from a Mn^2+^-sensing riboswitch^42^ through TL using a minimal representative dataset. Depending on the availability of training data, users can therefore choose to analyze a new biomolecular system by training a fully initialized network, or by adapting our pre-trained AutoSiM by Transfer Learning.

A potential future direction is to apply explainable deep learning algorithms such as decision tree algorithms^43^ to SMFM time traces to better understand the basis of decisions made by the networks. Along these lines, one could characterize the basis of algorithm decisions using simulated data to represent a broad range of time trace dynamics, similar to the approach we used to evaluate the LSTM classifier for SiMREPS data (Supplementary Fig. 3). Another future direction is to apply end-to-end deep learning algorithms for raw SMFM movie analysis by combining convolutional layers for image recognition with recurrent layers for time series classification in a deep learning network^44^. Last, but not least, unsupervised learning algorithms such as autoencoder networks^45^ may be possible to use in SMFM to further reduce the bias and variance of manual trace sorting.

We anticipate that the examples presented in this work will catalyze widespread improvements in the speed, accuracy, and convenience of single-molecule data analysis based on deep learning approaches.

## Methods

### Source data

For single-channel data classification and validation, we used SiMREPS measurements from the detection of the EGFR T790M DNA sequence as described previously^7^, and the HMM analysis and kinetic filtering were performed as described in the original paper. The training set comprises 706 and 1478 traces from mutant-only and wild-type only experiments, respectively, and the independent test set comprises 5457 traces from experiments collected at a constant concentration of mutant DNA in a varying excess of wild-type sequence as described previously^7^. For two-channel data from smFRET measurements, we used a dataset consisting of over 29,395 FRET traces that were manually analyzed and segmented by four different users studying different systems: a DNA swinging arm^36^; a toehold-exchange-based DNA walker^37^; a preQ_1_ riboswitch^39^; and a paused transcriptional elongation complex containing a preQ_1_ riboswitch^38^. Notably, these four users employed slightly different selection criteria such as minimum trace duration, minimum signal intensity or signal-to-noise ratio, and tolerance for non-correlated changes in donor or acceptor fluorescence intensity. To generate more variety in the input dataset, we include an additional set of 5,000 simulated traces generated in MATLAB with the assumption of first-order transition kinetics between two states with FRET efficiencies randomly chosen from a uniform distribution on the interval [0, 1], as well as first-order photobleaching kinetics.

### Recursive neural network analysis

The RNN (LSTM) takes raw SMFM intensity versus time traces as input and produces either trace classifications (Accept or Reject) or segmentation labels (frame-by-frame Accept or Reject labels) as output. The structures of the networks for trace classification and segmentation are shown in Fig. 2b.

To increase the network’s ability to recognize local patterns in the input sequence data, we designed the sequence input layer to convert each raw trace with *T* intensity-versus-time datapoints of dimension $[1,T]$ into a two-dimensional array of dimension $[n_{{\mathrm{bin}}},T/n_{{\mathrm{bin}}}]$, with each group of $n_{{\mathrm{bin}}}$ consecutive data points binned together. We found $n_{{\mathrm{bin}}} = 10$ to produce the best training results. If $n_{{\mathrm{bin}}}$ is too small, the information within each bin is insufficient for feature detection in the presence of noise. If $n_{{\mathrm{bin}}}$ is too large, it yields a high-dimensional feature space that hinders training.

The LSTM layer is composed of 100 Bidirectional LSTM hidden cells because they enable later frames to influence classification of earlier frames, which is a useful ability for the model. For instance, to determine the position of the first photobleaching event for segmentation purposes, it is helpful to know whether the signal reappears later in the trace.

In the classification layer, each label of the input data is given a predefined weight $w_i$, where $i$ represents the label. We define a weighted cross entropy loss function

1 $$L = - \mathop {\sum }\limits_i \mathop {\sum }\limits_j w_iY_{ij}\log P_{ij},$$

where $P_{ij}$ is the predicted probability that trace $j$ has label $i$, $Y_{ij} = 1$ if trace $j$ has label $i$, and $Y_{ij} = 0$ otherwise. In SiMREPS, we set $w_{{\mathrm{MUT}}} = 1$ and $w_{{\mathrm{WT}}} = 10^6$ in order to strongly penalize any false positive detection of the MUT sequence in the presence of only WT molecules. In smFRET trace classification, we set $w_{{\mathrm{accepted}}} = 1$ and $w_{{\mathrm{rejected}}} = 1$ in order to optimize the concordance between the network prediction and the human selection. In smFRET trace segmentation, we set $w_{{\mathrm{accepted}}} = 10$ and $w_{{\mathrm{rejected}}} = 1$ in order to increase the weight of manually selected segments in training, as these segments only represent a small fraction of the entire dataset (which contains many data frames after photobleaching of one or both fluorophores, and is therefore excluded by the user).

### Simulation of accepted traces

Acceptable (high-quality) smFRET traces were simulated in MATLAB using a Monte Carlo approach that assumes (1) two FRET states with energy transfer efficiencies randomly chosen from a uniform distribution between the limits of 0 and 1, and (2) single-exponential kinetics of transition between these states as well as single-exponential photobleaching kinetics. The rate constant of photobleaching for each fluorophore was assumed to be 0.0025 per frame (0.025 s^−1^), while the rate constants for transitions between FRET states were randomly chosen from an exponential distribution with average value 0.05 per frame (0.5 s^−1^). The mean total fluorescence intensity of a single donor or acceptor fluorophore (at FRET efficiency of 0 or 1, respectively) was assumed to be 1000 AU, and Gaussian noise with a standard deviation corresponding to varying fractions of the total signal was added to each simulated trace.

### Convolutional neural network analysis

In addition to the RNN, we deployed a CNN as an independent approach to smFRET trace classification. The structure of the network is shown in Fig. 2b. In this pipeline, raw traces are first converted into two-dimensional images by plotting the framewise donor and acceptor intensity values along the x- and y-axes of a scatter plot, respectively. The images thus show any positive or negative correlation between donor and acceptor intensities throughout the trace while not explicitly considering temporal dynamics. We used binning to down-sample the scatter plots to $32 \times 32$ pixels in order to reduce feature space and computational cost. Each image is associated with a label of either Accepted or Rejected by the human user. For our dataset, we found that training the network for ~5–10 h on a modern CPU is sufficient. On a GPU, we are able to achieve convergence within 1 h using NVIDIA’s TESLA V100. The trained network classifies SMFM traces at a rate of ~8000 min^−1^ using the CPU on a personal desktop with 3.4 GHz quad-core Intel Core i5 processor and 24 GB memory. A TL module is implemented to permit users to supplement the training set with their own data without completely retraining the network.

### Code implementation

The results presented in this paper are implemented using MATLAB 2018 with its Neural Network package. The networks are trained using the ADAM optimization method with an initial learning rate of 10^−4^. The parameters in the networks are initialized using the GLOROT random initializer.

### Reporting summary

Further information on research design is available in the Nature Research Reporting Summary linked to this article.

## Supplementary information

Supplementary Information

Reporting Summary

## Data Availability

The data that support the findings of this study are available at 10.7302/ck2m-qf69.

## Code availability

The source code of AutoSiM and the custom MATLAB scripts for the study in this paper is available at 10.7302/ck2m-qf69.
