# Supplementary material for: Automatic classification and segmentation of single-molecule fluorescence time traces with deep learning
Source: Nat Commun. 2020 Nov 17;11:5833. doi: 10.1038/s41467-020-19673-1 (PMC7673028; doi:10.1038/s41467-020-19673-1)
Supplement: Supplementary file 1 — Supplementary Information [file 41467_2020_19673_MOESM1_ESM.pdf]

SUPPLEMENTARY INFORMATION

Automatic classification and segmentation of single-molecule fluorescence time traces with deep learning

Jieming Li †[1,4], Leyou Zhang†[2,5], Alexander Johnson-Buck[1,3*], and Nils G. Walter[1*]

[1]Single Molecule Analysis Group, Department of Chemistry, The University of Michigan, Ann Arbor, MI, USA

[2]Department of Physics, The University of Michigan, Ann Arbor, MI, USA

[3]Department of Internal Medicine, Division of Hematology/Oncology, University of Michigan, Ann Arbor, MI, USA

[4]Current Address: Bristol-Myers Squibb, New Brunswick, NJ, USA

[5]Current Address: Google, Pittsburgh, PA, USA

†These authors contributed equally to this work

*nwalter@umich.edu, alebuck@med.umich.edu

a

	Selected Segments	Time (min)
**User 1**		
Condition 1	105	152
Condition 2	94	200
Condition 3	71	115
**User 2**		
Condition 1	233	60
Condition 2	359	61
Condition 3	223	46
**User 3**		
Condition 1	254	263
Condition 2	328	252
Condition 3	262	224
**User 4**		
Condition 1	171	163
Condition 2	172	246

b

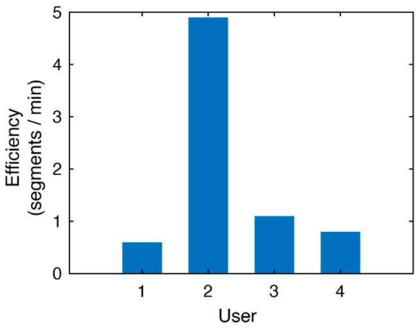

c

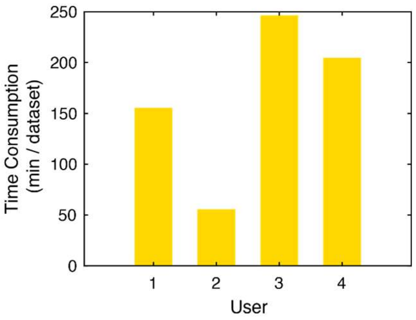

Supplementary Fig. 1 | Analysis of the time required for single-molecule dataset analysis by four researchers in the same laboratory. **a,** Table showing the number of single-molecule traces (segments) chosen and time spent on selection for each user and dataset (condition). The time required for analysis of each dataset was calculated from the timestamps of automatically saved segment files. **b**, Average number of segments selected per minute by different users. **c**, Average time needed by different users to complete manual selection of segments from one experimental dataset.

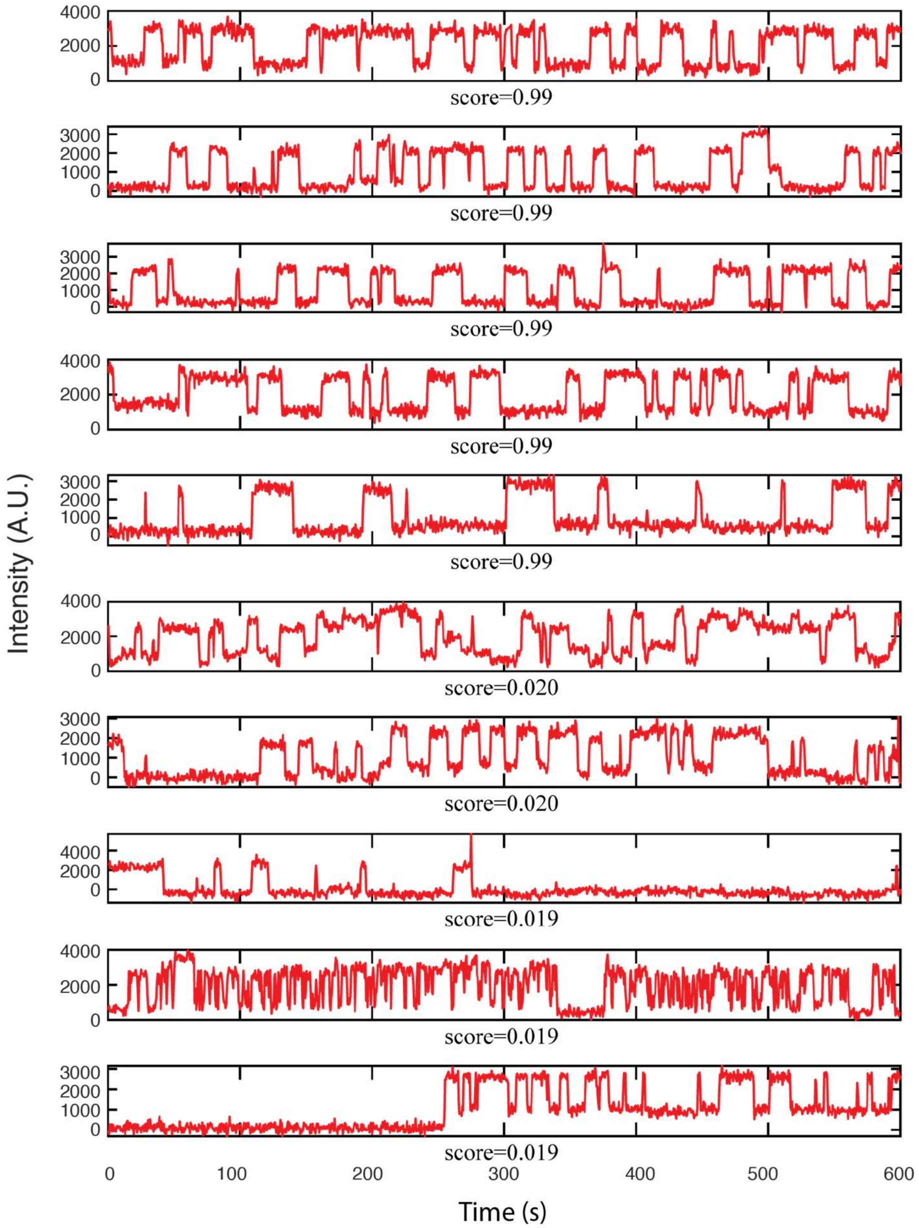

Supplementary Fig. 2 | Representative traces with high (>0.5) and low (≤ 0.5) scores assigned by the LSTM classifier of AutoSiM. The traces with low scores typically exhibit high variation in the baseline or kinetics that are not characteristic of true positives.

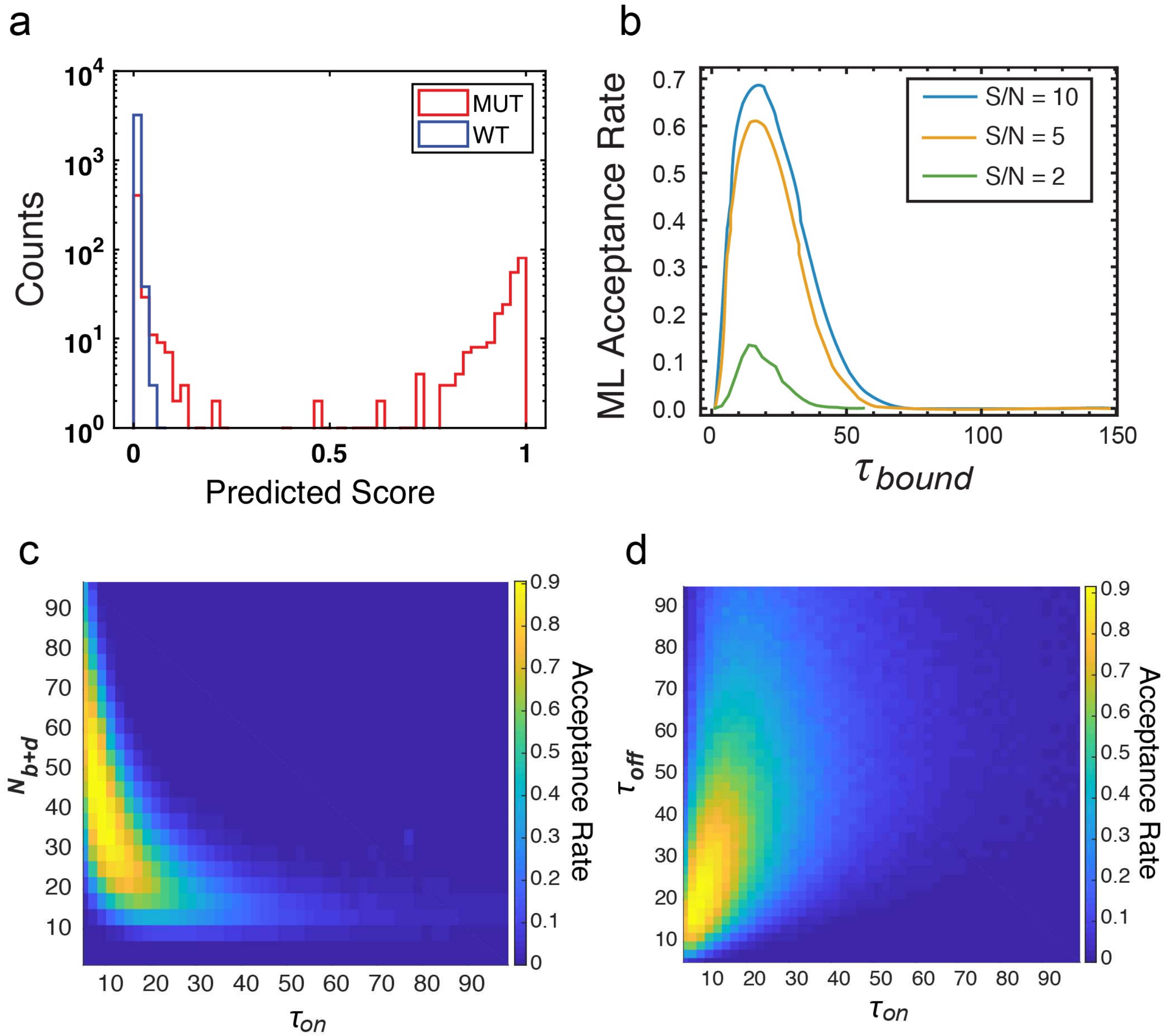

Supplementary Fig. 3 | Validation of trace classification by the LSTM algorithm of AutoSiM using experimental and simulated traces. **a,** Score distribution from LSTM classification of training data from mutant (MUT) and wild-type (WT) DNA control experiments. **b,** τ_{bound} distribution of simulated traces accepted by LSTM classification as a function of signal-to-noise (S/N). Traces having similar levels of signal-to-noise (S/N = 10, 5) to the traces used in training (average S/N = 6.7) are accepted with higher probability than simulated traces with lower signal-to-noise (S/N=2). **c, d,** Probability density maps showing the likelihood that the LSTM classification accepts simulated traces with different values of kinetic parameters N_{b+d} (number of binding and dissociation events), τ_{bound}, and $\tau_{unbound}$. Traces with similar kinetics to the training set are preferentially accepted despite the lack of an explicit kinetic model in the LSTM algorithm.

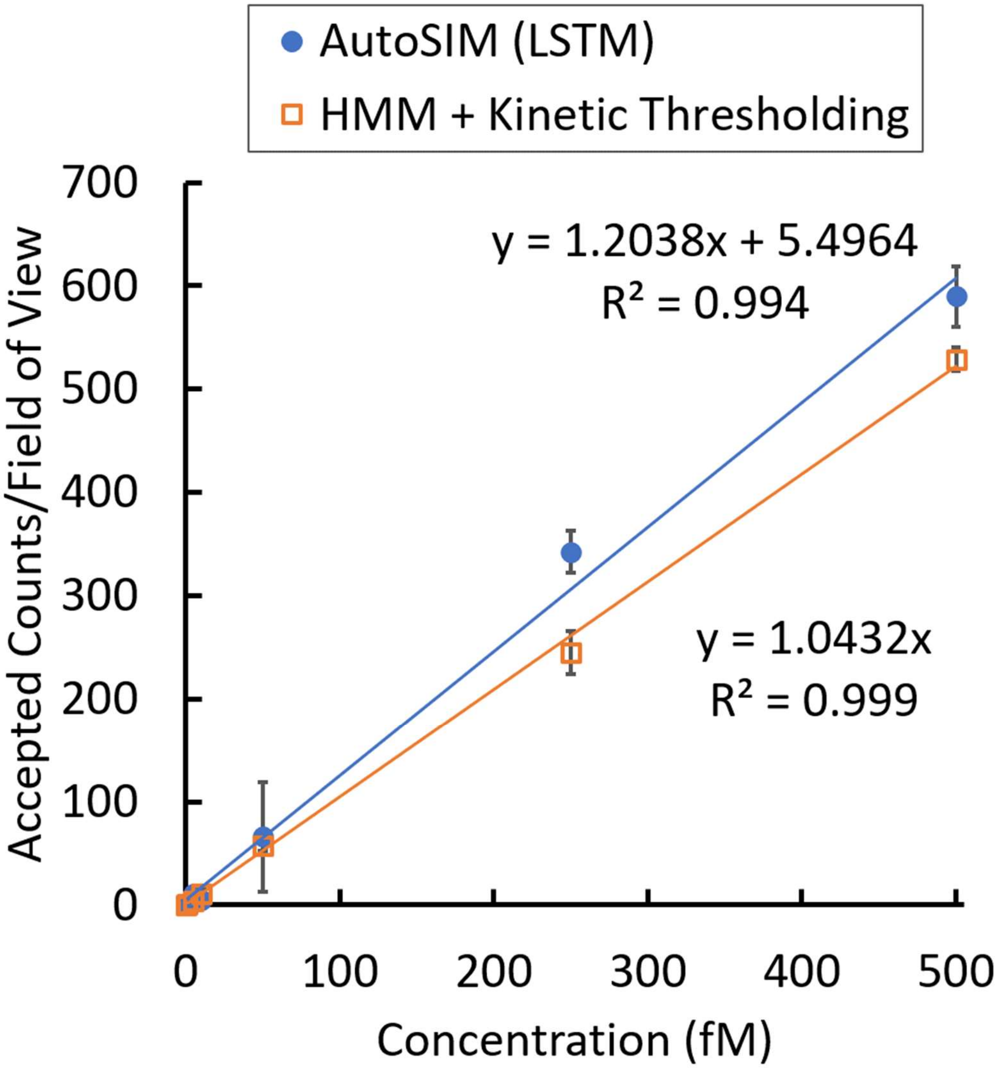

Supplementary Fig. 4 | Standard curve comparisons between LSTM of AutoSiM (blue) and HMM combined with kinetic thresholding (orange) for experiments containing a varying concentration of *EGFR* T790M (mutant) sequence, and no wild-type *EGFR* sequence. The LSTM analysis yields an approximately 15% increase in sensitivity. The error bars represent one standard deviation from three independent replicate measurements.

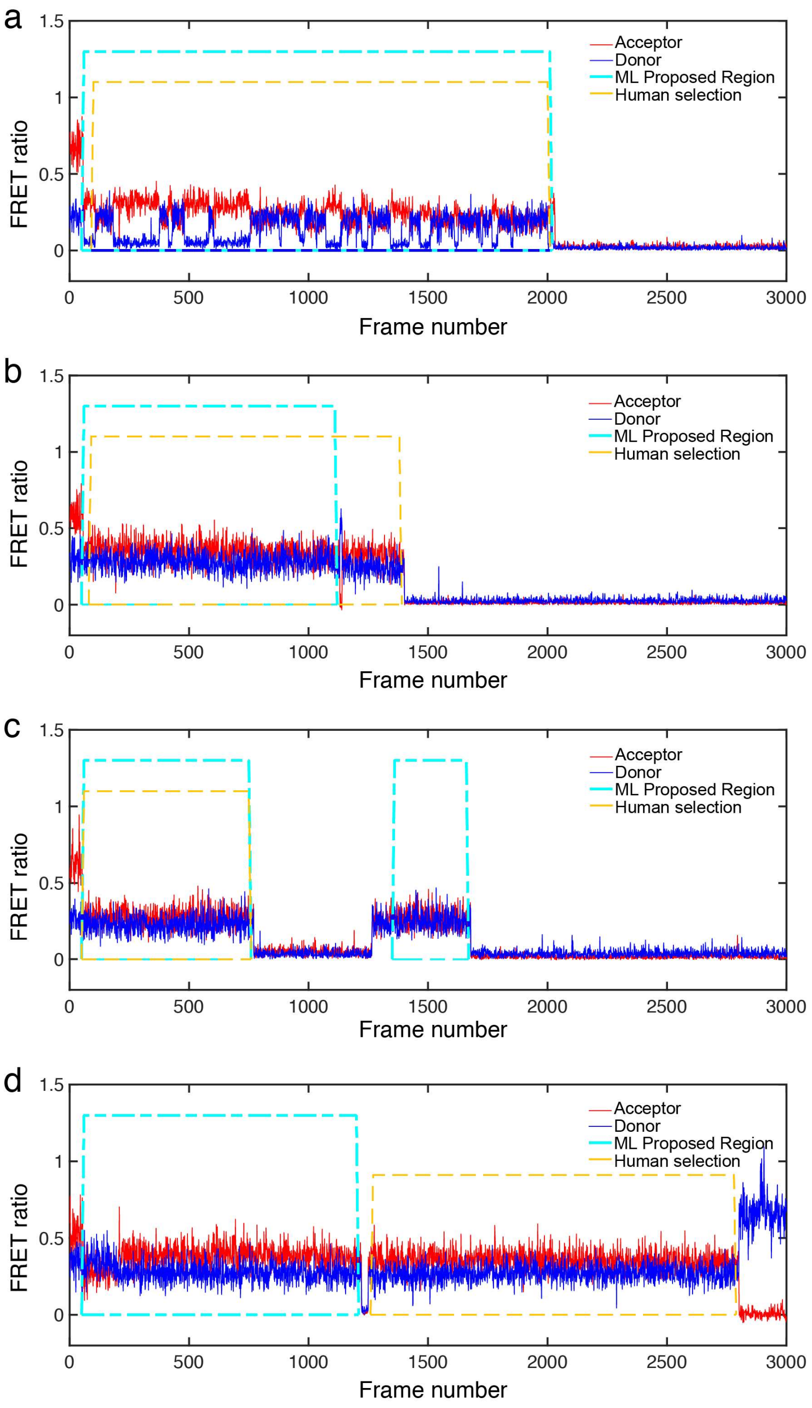

Supplementary Fig. 5 | Representative examples of segmentation of smFRET traces performed by human experimenters (yellow dashed lines) and the LSTM segmentation algorithm of AutoSiM (cyan dashed lines). **a**, In most cases, the machine learning algorithm selects almost the same region as human selection. **b**, In other cases, the human selection covers more frames than the machine learning algorithm because the algorithm is sensitive to sharp signal changes such as by photoblinking. **c**, In still other cases, the human experimenter selected only one of two possible contiguous spans of data, while the machine learning algorithm selects both. **d**, In rare cases, the LSTM algorithm and human experimenters' selections do not overlap at all, but both regions may be equally legitimate for FRET analysis.

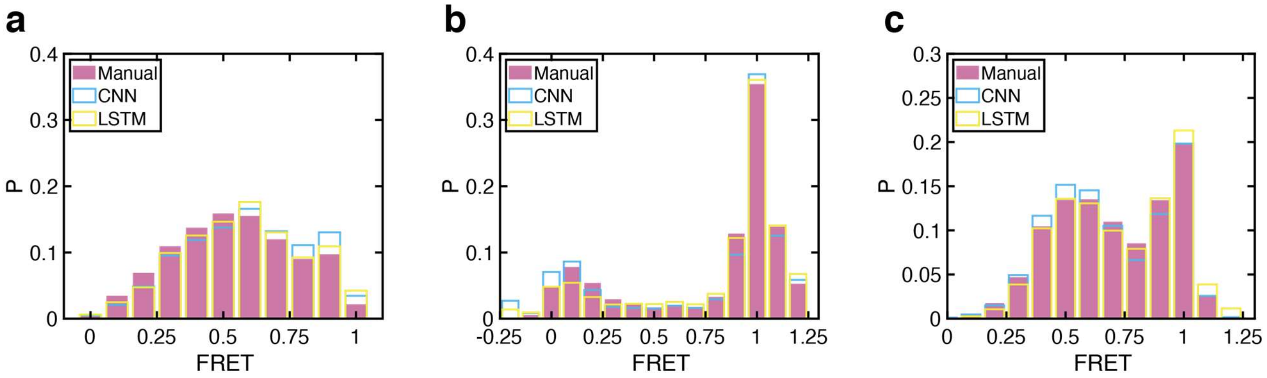

Supplementary Fig. 6 | FRET histograms generated by CNN-classified, LSTM-classified, and manually selected traces from the same two original datasets from 3 different experiment systems: **a,** a toehold-exchange-based DNA walker; **b,** a DNA swinging arm; **c**, a preQ1 riboswitch.

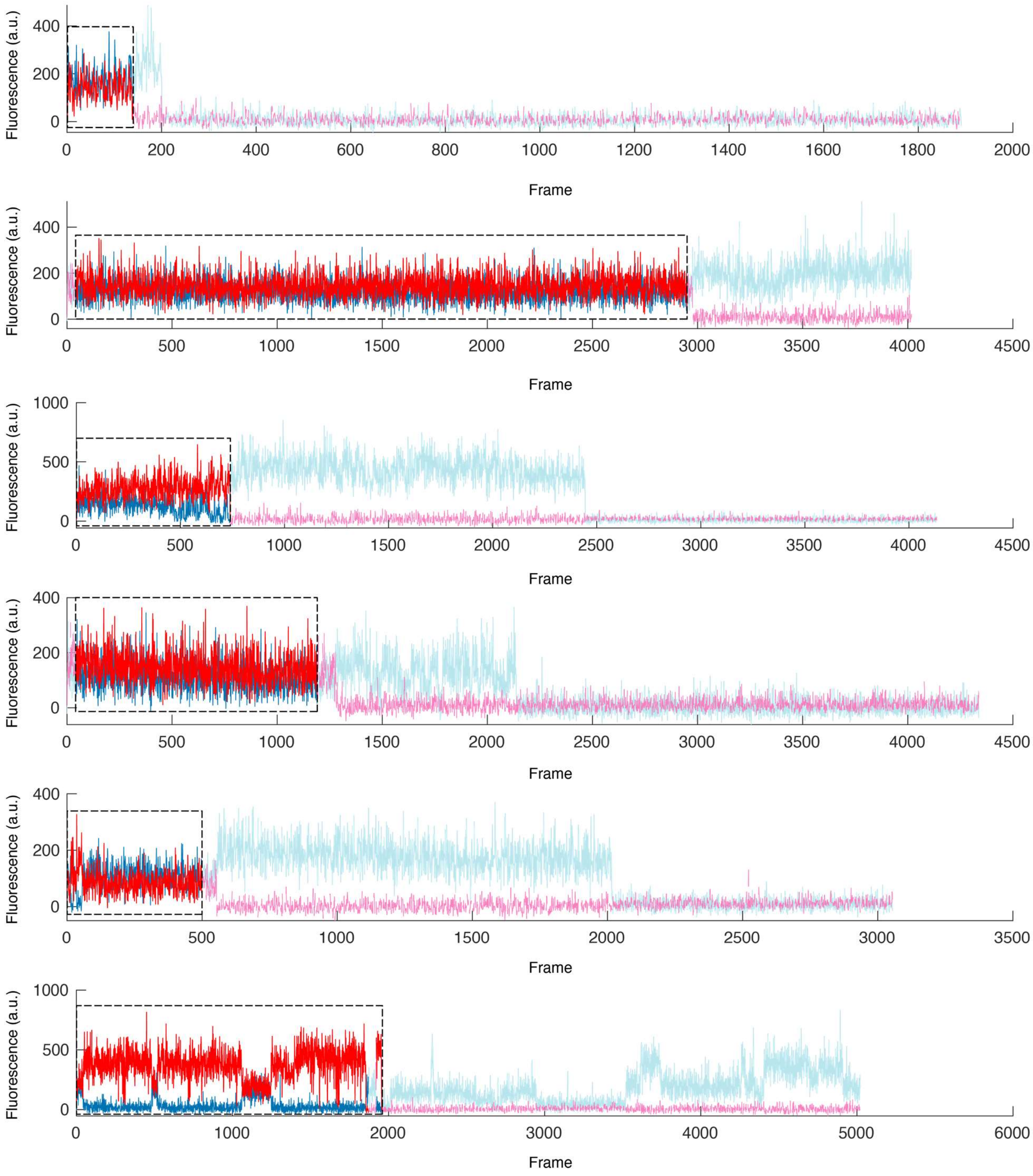

Supplementary Fig. 7 | Example traces that LSTM of AutoSiM accepted but manual selection did not.

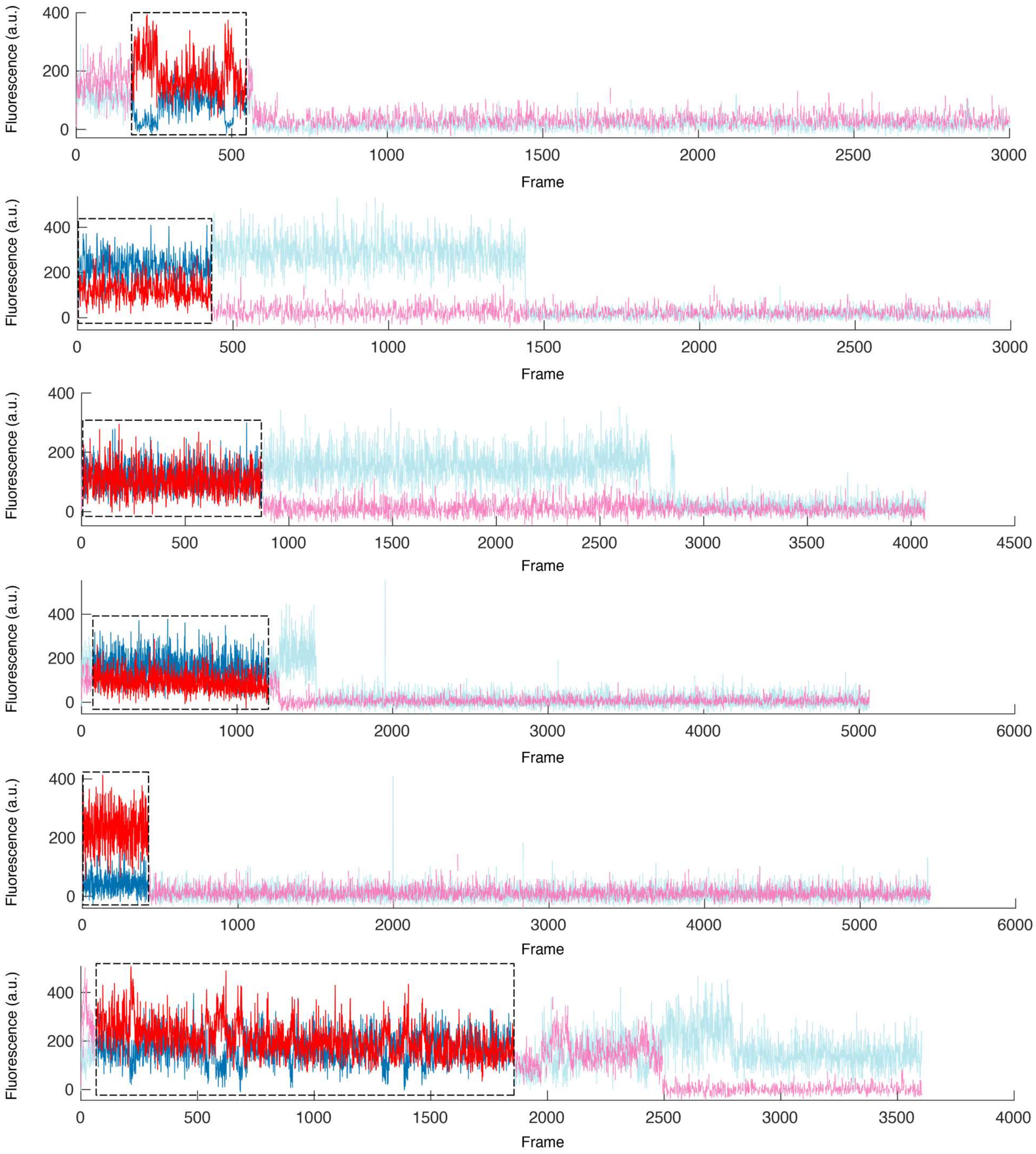

Supplementary Fig. 8 | Trace examples that manual selection accepted but LSTM did not.

Supplementary Table 1 | Concordance results from 10 independent experiments (training and test) with LSTM as implemented in AutoSiM. The training data set contained simulation traces.

Exp. #	Sensitivity	Specificity	Precision	Concordance
1-1	72.0%	95.2%	76.6%	91.1%
1-2	73.1%	94.4%	73.7%	90.6%
1-3	74.0%	93.6%	71.2%	90.2%
1-4	66.6%	94.7%	72.4%	89.8%
1-5	63.0%	96.7%	80.1%	91.0%
1-6	57.8%	95.0%	72.3%	88.2%
1-7	68.4%	94.1%	70.3%	89.8%
1-8	76.5%	94.6%	75.4%	91.4%
1-9	76.4%	93.5%	72.0%	90.3%
1-10	72.0%	93.8%	71.5%	90.0%
**Average**	**70.0%**	**94.6%**	**73.6%**	**90.2%**
**SD**	**6.0%**	**0.9%**	**3.0%**	**0.9%**

Supplementary Table 2 | Concordance results from 10 independent experiments (training and test) with LSTM as implemented in AutoSiM. The training data set did not contain any simulation traces.

Exp. #	Sensitivity	Specificity	Precision	Concordance
2-1	74.3%	93.7%	72.0%	90.2%
2-2	75.3%	94.2%	73.5%	90.9%
2-3	73.3%	95.9%	79.2%	91.9%
2-4	70.6%	96.2%	79.4%	91.7%
2-5	76.9%	94.8%	75.3%	91.7%
2-6	65.8%	96.4%	80.2%	90.8%
2-7	79.2%	93.0%	69.7%	90.6%
2-8	76.8%	94.4%	74.9%	91.3%
2-9	58.7%	97.0%	81.0%	90.1%
2-10	69.0%	95.6%	77.0%	90.9%
**Average**	**72.0%**	**95.1%**	**76.2%**	**91.0%**
**SD**	**6.2%**	**1.3%**	**3.8%**	**0.6%**

Supplementary Table 3 | Concordance results from 10 complete trials (training and testing on different randomly selected subsets of smFRET data) with CNN as implemented in AutoSiM. The training data set contained simulation traces.

Exp. #	Sensitivity	Specificity	Precision	Concordance
1-1	75.6%	93.9%	73.0%	90.7%
1-2	66.6%	95.4%	75.4%	90.3%
1-3	74.2%	94.3%	73.5%	90.8%
1-4	72.6%	94.8%	74.4%	90.9%
1-5	81.0%	93.2%	71.1%	91.1%
1-6	72.2%	95.1%	76.8%	90.9%
1-7	72.2%	94.4%	72.6%	90.7%
1-8	70.6%	94.3%	72.9%	90.1%
1-9	75.2%	94.6%	75.1%	91.1%
1-10	72.3%	94.6%	74.4%	90.7%
**Average**	**73.3%**	**94.5%**	**74.0%**	**90.7%**
**SD**	**3.7%**	**0.6%**	**1.6%**	**0.3%**

Supplementary Table 4 | Concordance results from 10 complete trials (training and testing on different randomly selected subsets of smFRET data) with CNN as implemented in AutoSiM. In these trials, the training dataset did not contain any simulated traces.

Exp. #	Sensitivity	Specificity	Precision	Concordance
2-1	73.5%	94.6%	74.8%	90.8%
2-2	75.9%	93.9%	72.9%	90.8%
2-3	75.2%	95.0%	75.8%	91.2%
2-4	73.0%	94.5%	73.5%	90.8%
2-5	73.2%	94.5%	73.4%	90.9%
2-6	80.1%	93.5%	73.5%	91.1%
2-7	77.0%	93.8%	71.5%	90.9%
2-8	66.0%	96.5%	80.5%	91.1%
2-9	70.4%	95.1%	75.9%	90.7%
2-10	76.2%	93.8%	72.7%	90.7%
**Average**	**74.1%**	**94.5%**	**74.5%**	**90.9%**
**SD**	**3.9%**	**0.9%**	**2.5%**	**0.2%**

Supplementary Table 5 | Concordance results from 10 complete trials (training and testing on different randomly selected subsets of smFRET data) with the intersection of LSTM and CNN as implemented in AutoSiM. In these trials, the training dataset contained simulated traces.

Exp. #	Sensitivity	Specificity	Precision	Concordance
1-1	61.8%	98.0%	86.8%	91.4%
1-2	56.1%	98.1%	86.5%	90.7%
1-3	60.5%	97.5%	83.5%	91.0%
1-4	54.0%	98.1%	85.4%	90.5%
1-5	56.8%	98.4%	88.1%	91.3%
1-6	47.8%	98.2%	85.6%	88.9%
1-7	55.4%	97.5%	81.8%	90.4%
1-8	59.6%	97.6%	84.5%	90.9%
1-9	63.0%	97.5%	84.7%	91.3%
1-10	59.1%	97.8%	85.5%	91.0%
**Average**	**57.4%**	**97.9%**	**85.2%**	**90.7%**
**SD**	**4.4%**	**0.3%**	**1.8%**	**0.7%**

Supplementary Table 6 | Concordance results from 10 complete trials (training and testing on different randomly selected subsets of smFRET data) with the intersection of LSTM and CNN as implemented in AutoSiM. In these trials, the training dataset did not contain any simulated traces.

Exp. #	Sensitivity	Specificity	Precision	Concordance
2-1	61.6%	97.7%	85.0%	91.2%
2-2	62.8%	97.6%	84.8%	91.5%
2-3	60.3%	98.0%	86.6%	91.4%
2-4	58.4%	98.1%	86.4%	91.2%
2-5	62.1%	97.7%	85.0%	91.6%
2-6	58.7%	97.9%	86.3%	90.7%
2-7	66.2%	96.8%	80.9%	91.6%
2-8	56.5%	98.4%	88.3%	89.9%
2-9	47.1%	98.8%	89.4%	89.5%
2-10	59.2%	97.9%	86.0%	91.1%
**Average**	**59.3%**	**97.9%**	**85.9%**	**91.0%**
**SD**	**5.1%**	**0.5%**	**2.3%**	**0.7%**

Supplementary Table 7 | Results for the TL-trained LSTM classification network of AutoSiM tested on smFRET data collected for the Mn^{2+}-sensing riboswitch under various experimental conditions. Tests 1, 2, 3, and 5 use data collected from different experimental conditions than the training data. Test 4 uses data from the same experimental condition as the training data, but collected independently.

#	Exp. Condition	Sensitivity	Specificity	Precision	Concordance
1	100 mM KCl, 0.1 mM EDTA	34.5%	97.7%	51.7%	93.59%
2	100 mM KCl, 0.5 mM Mg^{2+}	31.0%	97.9%	69.1%	88.85%
3	100 mM KCl, 1 mM Mg^{2+}	52.3%	97.0%	76.2%	90.01%
4	100 mM KCl,1 mM Mg^{2+},0.1 mM Mn^{2+}	51.3%	95.1%	57.2%	90.08%
5	100 mM KCl, 0.1 mM Mn^{2+}	45.6%	96.3%	47.5%	92.91%
	**Average**	**42.94%**	**96.80%**	**60.34%**	**91.09%**

Supplementary Table 8 | Results from automatic curation of smFRET traces from test data for the Mn^{2+}-sensing riboswitch with SPARTAN, for comparison with results from the LSTM network on the same dataset (Supplementary Table 7).

#	Exp. Condition	Sensitivity	Specificity	Precision	Concordance
1	100 mM KCl, 0.1 mM EDTA	64.4%	90.6%	32.4%	88.9%
2	100 mM KCl, 0.5 mM Mg2+	61.0%	91.1%	51.6%	87.1%
3	100 mM KCl, 1 mM Mg2+	76.5%	82.0%	44.0%	81.1%
4	100 mM KCl,1 mM Mg2+,0.1 mM Mn2+	65.0%	88.3%	40.6%	85.7%
5	100 mM KCl, 0.1 mM Mn2+	65.6%	89.7%	31.7%	88.1%
	**Average**	**66.50%**	**88.34%**	**40.06%**	**86.18%**

Supplementary Table 9 | Estimated time requirements for manual selection of time traces following automated curation with SPARTAN. (We assume manual selection has an average speed of 5 traces per minute.)

#	Exp. Condition	Total Traces # From Movie	# of Traces Rejected by Curation	# of Traces Need Manual Classification	Manual Time Usage* (min)
1	100 mM KCl, 0.1 mM EDTA	1326	1153	173	34
2	100 mM KCl, 0.5 mM Mg2+	1560	1312	248	50
3	100 mM KCl, 1 mM Mg2+	981	715	266	53
4	100 mM KCl,1 mM Mg2+,0.1 mM Mn2+	1430	1179	251	50
5	100 mM KCl, 0.1 mM Mn2+	1849	1590	259	52
	**Total**	7146	5949	1197	**239**

Supplementary Table 10 | Thresholds used for curation of smFRET traces with SPARTAN.

Feature	Thresholds
FRET at first frame	<1.00
Donor lifetime	>50.00
SNR-signal	>2.00
SNR-bg/SNR-signal	>1.50
Mean acceptor intensity	>60.00
Mean donor intensity	>30.00
FRET Lifetime	>25.00
D/A correlation	>-1.00
D/A correlation	<0.20
Signal-to-noise	>7.00
Background Noise	<250.00
# Cy3 blinks	<40.00

Supplementary Table 11 | Implementation details of the LSTM classification network of AutoSiM.

Layer Index	Type	Activation	Dimension Learnable Parameters
1	Sequence Input	20	--
2	Bi-Directional LSTM	200	Input Weights: 800×20, Recurrent Weights: 800×100, Bias: 800×1
3	Fully Connected	25	Weights: 25×200, Bias: 25×1
4	ReLU	25	--
5	Fully Connected	2	Weights: 2×25, Bias: 2×1
6	Softmax	2	--
7	Classification Output	--	--

Supplementary Table 12 | Implementation details of the LSTM segmentation network of AutoSiM.

Layer Index	Type	Activation	Dimension Learnable Parameters
1	Sequence Input	20	--
2	Bi-Directional LSTM	200	Input Weights: 800×20, Recurrent Weights: 800×100, Bias 800×1
3	Bi-Directional LSTM	200	Input Weights 800×200, Recurrent Weights 800×100, Bias 800×1
4	Fully Connected	25	Weights 25×200, Bias 25×1
5	ReLU	25	--
6	Fully Connected	2	Weights 2×25, Bias 2×1
7	Softmax	2	--
8	Segmentation Output	--	--

Supplementary Table 13 | Implementation details of the CNN classification network of AutoSiM.

Layer Index	Type	Kernel Size	Stride	Activations	Dimension Learnable Parameters
1	Image Input	--	--	32×32×3	--
2	Convolution	5×5×3	1×1	32×32×50	Weights 5×5×3×50, Bias 1×1×50
3	Batch Normalization	--	--	32×32×50	Offset 1×1×50, Scale 1×1×50
4	Max Pooling	3×3	2×2	15×15×50	--
5	ReLU	--	--	15×15×50	--
6	Dropout	--	--	15×15×50	--
7	Convolution	3×3×50	1×1	15×15×10	Weights 3×3×50×10, Bias 1×1×10
8	Max Pooling	3×3	2×2	7×7×10	--
9	ReLU	--	--	7×7×10	--
10	Convolution	3×3×10	1×1	7×7×10	Weights 3×3×10×10, Bias 1×1×10
11	ReLU	--	--	7×7×10	--
12	Convolution	3×3×10	1×1	7×7×10	Weights 3×3×10×10, Bias 1×1×10
13	Max Pooling	3×3	2×2	3×3×10	--
14	ReLU	--	--	3×3×10	--
15	Fully Connected	--	--	1×1×50	Weights 50×90, Bias 50×1
16	ReLU	--	--	1×1×50	--
17	Fully Connected	--	--	1×1×2	Weights 2×50, Bias 2×1
18	Softmax	--	--	1×1×2	--
19	Classification Output	--	--	--	--
