# Supplementary material for: Automatic classification and segmentation of single-molecule fluorescence time traces with deep learning
Source: Nat Commun. 2020 Nov 17;11:5833. doi: 10.1038/s41467-020-19673-1 (PMC7673028; doi:10.1038/s41467-020-19673-1)
Supplement: Supplementary file 2 — Reporting Summary [file 41467_2020_19673_MOESM2_ESM.pdf]

nature research

Corresponding author(s): Nils G. Walter, Alexander Johnson-Buck

Last updated by author(s): 10/9/2020

Reporting Summary

Nature Research wishes to improve the reproducibility of the work that we publish. This form provides structure for consistency and transparency in reporting. For further information on Nature Research policies, see Authors & Referees and the Editorial Policy Checklist .

Statistics

For all statistical analyses, confirm that the following items are present in the figure legend, table legend, main text, or Methods section.

n/a	Confirmed	
☐	☒	The exact sample size (*n*) for each experimental group/condition, given as a discrete number and unit of measurement
☐	☒	A statement on whether measurements were taken from distinct samples or whether the same sample was measured repeatedly
☒	☐	The statistical test(s) used AND whether they are one- or two-sided<br>*Only common tests should be described solely by name; describe more complex techniques in the Methods section.*
☒	☐	A description of all covariates tested
☒	☐	A description of any assumptions or corrections, such as tests of normality and adjustment for multiple comparisons
☐	☒	A full description of the statistical parameters including central tendency (e.g. means) or other basic estimates (e.g. regression coefficient) AND variation (e.g. standard deviation) or associated estimates of uncertainty (e.g. confidence intervals)
☒	☐	For null hypothesis testing, the test statistic (e.g. *F*, *t*, *r*) with confidence intervals, effect sizes, degrees of freedom and *P* value noted<br>*Give P values as exact values whenever suitable.*
☒	☐	For Bayesian analysis, information on the choice of priors and Markov chain Monte Carlo settings
☒	☐	For hierarchical and complex designs, identification of the appropriate level for tests and full reporting of outcomes
☒	☐	Estimates of effect sizes (e.g. Cohen's *d*, Pearson's *r*), indicating how they were calculated

Our web collection on statistics for biologists contains articles on many of the points above.

Software and code

Policy information about availability of computer code

Data collection	Data were collected using custom MATLAB code as described in the original studies from which the data originate.
Data analysis	Data analysis was performed using custom MATLAB Version 9.6 (R2019a; Statistics and Machine Learning Toolbox Version 11.5 Deep Learning Toolbox Version 12.1) code as described in the original studies from which the data originate; machine learning analysis was performed using custom MATLAB code written specifically for this study and described in the Methods section, and are available for download at https://doi.org/10.7302/ck2m-qf69, along with sample training and test data.

For manuscripts utilizing custom algorithms or software that are central to the research but not yet described in published literature, software must be made available to editors/reviewers. We strongly encourage code deposition in a community repository (e.g. GitHub). See the Nature Research guidelines for submitting code & software for further information.

Data

Policy information about availability of data

All manuscripts must include a data availability statement. This statement should provide the following information, where applicable:

- Accession codes, unique identifiers, or web links for publicly available datasets
- A list of figures that have associated raw data
- A description of any restrictions on data availability

The datasets analyzed during the current study are available from the corresponding author on reasonable request.

Field-specific reporting

Please select the one below that is the best fit for your research. If you are not sure, read the appropriate sections before making your selection.

Life sciences study design

All studies must disclose on these points even when the disclosure is negative.

Sample size	Sample sizes are indicated in the main text and Methods section. For the single-channel classification, the training set comprises 2,184 single-molecule traces, and the independent test set comprises 5,457 traces. For the two-channel classification and segmentation, the dataset comprises 29,395 single-molecule traces and (optionally) 5,000 additional simulated traces. The sample sizes were chosen based on availability of data, but are within or above the norms for the field of single-molecule biophysics, and yield reasonably reproducible results upon randomized subdivision of the data into different training and test sets (see Supplementary Tables 1-6).
Data exclusions	No data were excluded from this analysis.
Replication	Single-channel datasets were taken from experiments in which three independent replicate measurements were taken under each condition, as described in the original study (ref 18). While the two-channel studies used the same underlying datasets, to assess reproducibility of the algorithms developed in this work we randomly divided the same datasets repeatedly into different subsets for training and testing of the classifier algorithms, respectively. This was done 10 times for each set of conditions tested, and the results reported in Supplementary Tables 1-6. All replicate attempts were successful and no replicates were discarded.
Randomization	Sets of single-molecule traces for classification were subdivided into training and test sets at random.
Blinding	Allocation of data to training and test sets was performed randomly by computer algorithm and not supervised by the experimenters. However, no formal blinding was used because the subsequent training and classification were performed by computer algorithm.

Reporting for specific materials, systems and methods

We require information from authors about some types of materials, experimental systems and methods used in many studies. Here, indicate whether each material, system or method listed is relevant to your study. If you are not sure if a list item applies to your research, read the appropriate section before selecting a response.

Materials & experimental systems

n/a	Involved in the study	
☒	☐	Antibodies
☒	☐	Eukaryotic cell lines
☒	☐	Palaeontology
☒	☐	Animals and other organisms
☒	☐	Human research participants
☒	☐	Clinical data

Methods

n/a	Involved in the study	
☒	☐	ChIP-seq
☒	☐	Flow cytometry
☒	☐	MRI-based neuroimaging
